# Successful Correction of Postinfarction Interventricular Septum Rupture Diagnosed Online During the COVID-19 Pandemic (Clinical Case)

**DOI:** 10.21470/1678-9741-2021-0492

**Published:** 2023

**Authors:** Nazgul Asylbekovna Seitmaganbetova, Khibina Mirshat, Veklenko Viktorovna Galina, Tleumagambetova Bibolatovna Bibigul, Aliyev Mynbayevich Ondasyn, Rakhmatullina Nikolayevna Tolkynai, Biyasilov Nurzhan, Zhaubatyrova Aigul, Kurmasheva Gulnara

**Affiliations:** 1 Department of Propaedeutics of Internal Diseases, Non-Profit Joint Stock Company “West Kazakhstan Medical University named after Marat Ospanov”, Aktobe, Kazakhstan.; 2 Therapeutic Department, The State Municipal Enterprise “Hospital of Emergency Medical Care” on the Right of Economic Management of the Health Department, Aktobe, Kazakhstan.

**Keywords:** Myocardium Infarctation, Ventricular Septum/diagnostic, COVID-19, Pandemic

## Abstract

Postinfarction interventricular septum defect is a rare, but very serious and sometimes fatal, complication of acute myocardial infarction. This article describes a clinical case of online diagnosis of a late-stage myocardial infarction and the subsequent successful endovascular repair of a postinfarction ventricular septum defect with a Myval™ occluder.

## INTRODUCTION

Postinfarction ventricular septal defect (VSD) is a rare complication of acute myocardial infarction (AMI) and is accompanied by high mortality due to the development and rapid progression of acute heart failure. The formation of a postinfarction defect usually happens within the first week after the occurrence of acute ischemia, mainly (94%) occurring within the first 16 hours, as confirmed and followed by the SHOCK (standing for SHould We Emergently Revascularize Occluded Coronaries for Cardiogenic ShocK) study^[1]^.

The active use of reperfusion therapy in the treatment of acute transmural infarction reduced the frequency of interventricular septal myocardial ruptures by 5-6 times according to various data — 0.5% of all cases of acute transmural myocardial infarction^[2-5]^ *vs.* 1-3% of cases without the use of a reperfusion treatment strategy. The duration of this complication has also changed — from 3-5 days, without any reperfusion therapy, to the first day, in the case of primary endovascular intervention or thrombolytic therapy^[6,7]^. In most cases (70%), a rupture of the interventricular septum (IVS) occurs as a complication of anterior transmural myocardial infarction. In 66% of cases, apical localization of the rupture is observed, and in 34%, basal localization.

It is known that the cohort of “increased risk of myocardial rupture” is an elderly contingent of female patients with diabetes mellitus, with the first (in anamnesis) anterior transmural myocardial infarction, and in the absence of reperfusion in the first 3-6 hours from the onset of the disease or receiving late drug or mechanical reperfusion^[4,6]^.

In the natural course of postinfarction VSD, about 24% of patients die within the first 24 hours, 50% within the first week, and 87% within six weeks^[8,9]^. Approximately 5 % of all AMI deaths are associated with this complication^[10,11]^.

We present a clinical case of late online diagnosis of an acute postinfarction defect of the IVS with subsequent successful surgical correction.

## CASE PRESENTATION

Patient C., 57 years old, was admitted to the clinic of the Cardiology Department of the SME “Hospital of Emergency Medical Care” on the Right of Economic Management of the Health Department of Aktobe region on August 28, 2020 with complaints of shortness of breath at the slightest physical exertion, cough with difficult-to-separate sputum, aching pains in the scapular region on the left side, swelling on both lower extremities, and general weakness.

From the medical case history, she was ill for about two months, and she was being treated in the Cardiology Department of the Medical Center of the West Kazakhstan Marat Ospanov State Medical University, from July 04 to July 15, 2020, for coronary heart disease (CHD). The patient had AMI of the lower wall of the left ventricle with capture of the right ventricle with a Q wave on July 03, 2020, a three-vessel lesion of the coronary bed. Stenting of the right coronary artery was done on July 04, 2020. Sequelae were Killip class 1 and ischemic hepatopathy. Background diseases were type 2 diabetes mellitus in stage of severe decompensation and chronic renal disease 3b (GFR CKD-EPI [2011] 53 ml/min on the background of diabetic nephropathy [contrast-induced diabetes]). She was discharged with improvement of her state, and it was recommended to continue dual antiplatelet therapy.

Results of echocardiography from July 15, 2020 before discharge from the Marat Ospanov Medical Center were compacted aortic wall and slightly enlarged left atrial cavity. There were also pumping and contractile functions of the left ventricle (52%), hypertrophy of the myocardium and of the anterior wall of the pancreas, diastolic dysfunction of both ventricles, mitral regurgitation, and tricuspid regurgitation (first heart sound) and estimate of the mean pulmonary artery pressure 31 mmHg.

**Fig. 1:**
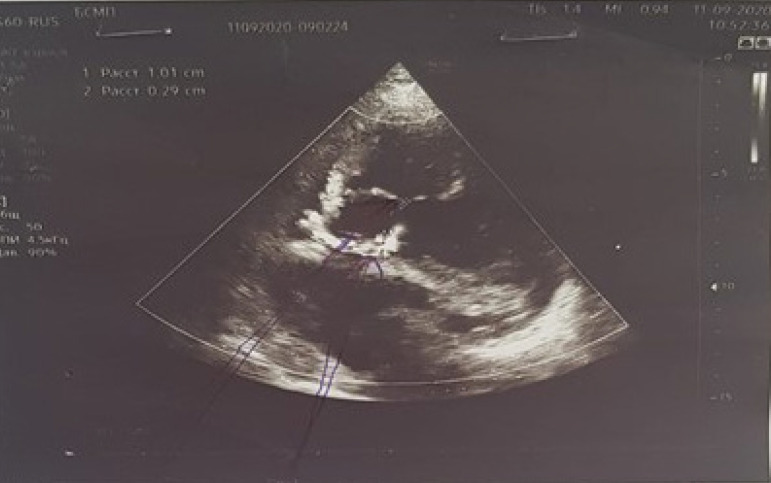
Echocardiography before surgery.

After discharging from the hospital on the next day, there was shortness of breath during physical exertion and general weakness. She went to the local therapist, a computerized tomography scan of the chest organs was performed, and it revealed lower and middle lobe segmental pneumonia of the right lung, exudative pleurisy mainly in the right pleural cavity, exudative pericarditis, cardiosclerosis, stenting of the coronary arteries, chronic obstructive bronchitis, and emphysema of the lungs. On an outpatient basis, she received antibacterial therapy. Despite the therapy, her health did not improve — shortness of breath increased at rest and on the slightest physical exertion, aching pains in both scapular areas, and edema appeared on both lower extremities. The patient again consulted the district therapist in the private clinic “Kuanysh”. A control X-ray of the chest organs was performed on July 24, 2020 and showed signs of exudative pleurisy with a fluid level from the IV rib to the diaphragm. The heart was expanded, and there was pneumosclerosis on the left. Ultrasound of the pleural cavities on July 28, 2020 concluded the presence of free fluid (about 2177 ml) located on the right. She was examined by a phthisiologist to rule out tuberculosis. With the diagnosis of right-sided pleurisy, she was sent for inpatient treatment to the SME “Hospital of Emergency Medical Care” on the Right of Economic Management of the Health Department of Aktobe region. Echocardiography was performed at the reception ward on September 01, 2020 —in the projection along the short axis of the mitral valve at 9 h and 10 h and in the four-chamber position, an interruption of the echo signal (1.2 cm × 0.3 cm) with a blood discharge from left to right was revealed. The aortic wall was thickened. The right parts, the left atrial cavity, and the trunk of the pulmonary artery were enlarged. There was also hypertrophy of the right ventricular myocardium. The global contractility and pumping function of the left ventricle were reduced (ejection fraction [EF] 50%). There were hypokinesis of the basal, middle anterior-septal, anterior, posterior, posterior-septal, and septal-apical segments. The contractility of the right ventricle was reduced (tricuspid annular plane systolic excursion [TAPSE] 1.4 cm). The diastolic function of the ventricles was preserved. The valve body was thickened, and significant separation of pericardial layer, minor pulmonary hypertension, and slightly elevated pulmonary pressure (37.43 mmHg) were estimated. There were also additional chord of the left ventricle, accelerated gradient on aortic valve 12.12 mmHg, and mild mitral regurgitation. Moderate tricuspid regurgitation, mild pulmonary regurgitation, and minimal aortic regurgitation were revealed (Figure 1).

The condition was of moderate severity due to the heart failure. The patient was conscious and correctly answered all questions. The constitution was normosthenic — height, 163 cm; weight, 77 kg; and body mass index, 28.98 kg/m2. The position in the bed was orthotic. Breathing through the nose was free. The chest shape was correct. Vesicular resonance in the lower parts was greater on the left. Auscultation on the left side indicated weakened breathing; on the right side, the breath was not heard (respiratory rate 24-26 breaths/minute). The area of the heart had no visible pathology. The apical push was palpable along the left mid-clavicular line, but it was weakened. The boundaries of the relative dullness of the heart were extended to the left. The heart tones were muted, the rhythm was correct, and there was a rough systolic noise at all points. Heart rate (HR) was 80 bpm, and blood pressure(BP) was 120/80 mmHg. Appetite was reduced. The tongue was moist and covered with a white coating. The abdomen was soft and painless on palpation. Liver was along the edge of the costal arch. Action of the bowels and diuresis was regular. Peripheral edema was on both lower extremities up to the knees and of dense consistency.

In laboratory studies, attention was drawn to a reduced level of hemoglobin (109 g/l), thrombocytopenia (150000), hyperglycemia (12 mmol/l), and hyperfermentemia (alanine aminotransferase 97,20000 IU/l). The remaining indicators were within normal range.

Ultrasound examination of the pleural cavities from September 01, 2020 showed free fluid (about 1100 ml) located in the right pleural cavity, and it was not located on the left. Conclusion was hydrothorax on the right. The pleural cavity was drained.

Chest X-ray was performed on September 03, 2020. In the image of the lungs, in the lower parts of the right side, there was an intense darkening associated with the root of the lung; the roots were intense, slightly structured, the shadow of the heart was expanded, and the sinuses were free. Conclusion was prolonged pneumonia on the right side, pneumosclerosis, and expansion of the shadow of the heart.

Electrocardiogram (ECG) showed sinus rhythm with HR of 63 bpm. There was Q-forming infarction of the lower wall of the myocardium; the prescription for ECG can be judged by the clinic, and the dynamics of the ECG are to be combined with the clinic.

According to the echocardiographic results, consulted online by Professor Akhmetov K. Zh., the diagnosis was made: “CHD. Post infarction cardiosclerosis (03.07.2020), Three-vessel lesion of the coronary bed, Stenting of the right coronary artery (04.07.2020), Postinfarction rupture of the interventricular septum. Concomitant disease: Type 2 diabetes mellitus, moderate severity in the case of subcompensation, Diabetic micro-macroangiopathy. Complication: HF (Heart Failure) Stage IIB”.

A cardiac surgeon was consulted on September 03, 2020, and the diagnosis was: “CHD. Postinfarction cardiosclerosis (03.07.2020 g), three-vessel lesion of the coronary bed. Stenting of the right coronary artery from 04.07.2020. Complication: Dressler's syndrome. Exudative pleurisy, pericarditis. Post infarction muscular VSD (ventricular septal defect). HF Stage IIB”. Accompanying disease was: “Type 2 diabetes mellitus, moderate severity in stage III and subcompensation. Diabetic micro-macroangiopathy. Chronic renal disease, stage 3 B GFR CKD-EPI”. It was recommended to send the patient to the National Scientific Cardiac Surgery Center (NSCSC) of the city of Nursultan for endovascular closure of the muscular VSD after stabilization of the condition.

The patient received the following treatment in the hospital: diuretics, glucocorticosteroids, anticoagulants, antibacterial therapy, beta-blockers, and angiotensin-converting enzyme inhibitors. At discharge, her health significantly improved, there was no cardiac decompensation, and she was discharged with the recommendations of endovascular closure of the muscular VSD in the NSCSC (Nursultan).

Four months (December 15, 2020) after the development of AMI, echocardiography was performed as planned in the NSCSC (Nursultan). At the level of the IVS postinfarction defect, with a size of 0.6×0.8 cm on the 3^rd^ segment, a shunt flow from left to right was registered. Through the ventricular gradient - 86 mmHg. The atrial septum is intact. There was dyskinesis of the 3, 4, 9, and 10 segments of the left ventricle with thinning to 0.4 cm. Also, separation of the pericardial leaves, along the posterior-lateral wall of the left ventricle (0.3 cm). Conclusion was postinfarction VSD, left ventricular (LV) aneurysm of lower localization, dilatation of the left parts and the right atrium, reduced global LV systolic function, LV myocardial hypertrophy, and mild mitral valve and tricuspid valve insufficiency.

On December 22, 2020, coronary angiography and probing (catheterization) of the heart cavities were performed, followed by closure of the VSD with Myval™ occluder.

Echocardiography from December 23, 2020 showed separation of pericardial layer: behind the lateral wall of the left ventricle (0.3 cm), along the posterior wall of the left ventricle (0.2 cm), and behind the anterior wall of the right ventricle (0.5 cm). In the IVS, an occluder is visualized, sealed, and there is a slight para-occluder peak.

On the 10^th^ day, the patient was dismissed from the hospital with recommendations for observation by the district therapeutic cardiologist.

One month (February 12, 2021) after endovascular closure of VSD, the patient’s condition is satisfactory, which is confirmed by the objective status and the results of echocardiography. There are no complaints, hemodynamics is stable. There is vesicular breathing above the lungs, no wheezing. BP is 120/80 mmHg. HR is 78 bpm. The rhythm is regular. A moderate systolic murmur is heard by at the heart apex. The abdomen is soft, painless. And there was no peripheral edema.

**Fig. 2:**
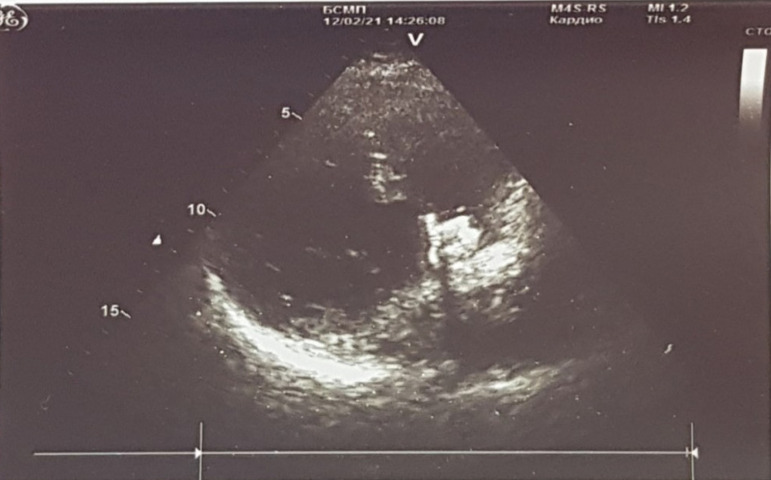
Echocardiography one month after surgery.

Echocardiography dated February 12, 2021 showed the condition after VSD closure with Myval™ occluder: the aortic wall was compacted. LV cavity was dilated. Pronounced eccentric hypertrophy of the LV myocardium. Global contractility and pumping function of the left ventricle were reduced (EF 52%). The basal posterior-septal segment of IVS was thinned, the occlude was visualized, and it was sealed. Contractility of the right ventricle was preserved (TAPSE-2.2 cm). Right ventricular diastolic dysfunction grade 1. The valve body was sealed. Minor separation of the pericardial layer. There were no septal defects. Minor pulmonary hypertension of reflex sympathetic dystrophy (32.11 mmHg). Mild mitral regurgitation. Tricuspid regurgitation of mild degree. Minimal pulmonary regurgitation. And there was no aortic regurgitation (Figure 2).

## DISCUSSION

A postinfarction defect of the IVS is most often formed in the acute phase of a heart attack. Within a few weeks, coagulation necrosis of the myocardium develops, due to lytic enzymes, and the necrotic myocardium is distributed. The size of the defect increases due to continued necrosis, resorption, and retraction of necrotic tissue. In the subacute phase of infarction, the myocardium heals. The septum becomes more fibrotic, and scarring develops, which is why many surgeons prefer to postpone the operation for several weeks to ensure the correct apposition of the edges and suture attachment during the operation^[9-12]^. In the largest meta-analysis, Arnaoutakis et al. analyzed data from 2,876 patients undergoing surgical closure of a VSD. Mortality among them was 54.1% in the group up to seven days from myocardial infarction and 18.4% in the group of patients operated after this time. The longer the interval between myocardial infarction and surgery, the lower the probability of death (< 6 h, odds ratio [OR] 6.18; 6-24 h, OR 5.53; 1-7 days, OR 4.59; 8-21 days, OR 2.37; *P*<0.01)^[12]^. We believe that this pattern is valid for endovascular intervention, and the duration of the procedure depends mainly on the severity of the patient’s condition.

Timely detection of surgical complications of myocardial infarction and their correction remain the only method that can save the patient’s life. In our clinical case, the patient was discharged from the hospital on the 14th day after AMI in the absence of a defect and signs of Dressler syndrome according to ultrasound data. The clinic of cardiac decompensation and Dressler syndrome, which developed later, was regarded as outpatient pneumonia and pulmonary complications in a patient who received inpatient care during the quarantine conditions of the coronavirus disease 2019 pandemic. Conservative (antibacterial) therapy was ineffective, difficulties in performing echocardiography due to the pandemic led to the progression of cardiac decompensation and to emergency hospitalization in the SME “Hospital of Emergency Medical Care” on the Right of Economic Management of the Health Department of Aktobe region. Successful online diagnosis of postinfarction VSD and subsequent effective endovascular intervention, performed in a stable condition four months after the myocardial infarction and stenting of the right coronary artery, stabilized hemodynamics and significantly improved the quality of life of our patient.
